# Intractable headache with autonomic features after gamma knife radiosurgery: A case report

**Published:** 2019-07-06

**Authors:** Mansoureh Togha, Abdorreza Naser Moghadasi, Samaneh Haghighi, Mahmood Motamedi

**Affiliations:** 1 Department of Neurology, School of Medicine, Tehran University of Medical Sciences, Tehran, Iran; 2 Sina Hospital, Tehran University of Medical Sciences, Tehran, Iran

**Keywords:** Headache, Intractable, Autonomic Effect, Pituitary Neoplasms, Trigeminal Autonomic Cephalalgia, Cluster Headache

Hypophyseal tumors are most commonly seen in the area of the sella.^1^ Headache is a common feature of these tumors.^2^^,^^3^ Headache patterns of these tumors can mimic several primary headache disorders such as migraine, cluster, primary stabbing headache, and hemicrania continua.^4^

Different methods like transsphenoidal surgery and radiosurgery which are performed to treat these tumors can make the headaches worse or cause a new headache type.^5^^,^^6^

In a literature search, only one case of long-term headache was reported following transsphenoidal surgery^7^ which was in close relation to the surgery, and was different from our case in terms of headaches onset time. Here, we present a case of headaches with autonomic features 3 months after uncomplicated gamma knife radiosurgery of remnant hypophysial adenoma. She underwent transsphenoidal macroadenoma surgery 6 years prior to her gamma knife radiosurgery. During this long time, she did not have any complaint of headache, so the procedure may play a role in the development of the headaches.

In recent reports, some patients who underwent stereo-tactic surgery have been noted that their presurgical headaches exacerbated following the gamma knife radiosurgery.^8^

The patient was a 24-year-old woman with a past history of galactorrhea underwent transsphenoidal surgery for pituitary macroadenoma 6 years before. She had gamma knife radiosurgery 3 years later for the tumor remnant.

The patient developed new onset severe and strictly unilateral stabbing headaches associated, with left eye lacrimation and restlessness, 3 months after gamma knife radiosurgery.

Frequency of attacks was at least 3 times a day lasting 40-60 minutes episodically. Symptoms described by the patient seemed to be typical for a diagnosis of cluster headache according to the International Classification of Headache Disorders-3 (ICHD-3) criteria. Physical examination was normal. Laboratory tests were also normal including pituitary hormones. 

Indomethacin 225 mg daily, subcutaneous sumatriptan and oxygen during attacks, verapamil 480 mg daily, and lithium 900 mg daily all were unsuccessful in treating the patient’s headache attacks.

Unfortunately, within 2 years follow-up, she was resistant to all above-mentioned drugs, and also to full dose of pregabalin, lamotrigine, sodium valproate, botulinum toxin injections, and sphenopalatine ganglion, supraorbital, and greater occipital nerves blocks. She had only partial response to short-time high-dose prednisolone.

The sella turcica is located in the vicinity of several critical brain structures. Involvement of any of the structures can be accompanied by intense headaches. The headache is sometimes so severe, does not respond to drug therapy, and requires surgery.^9^

In our case, no prior history of headache was mentioned by the patient, and headaches developed following the second procedure i.e. gamma knife radiosurgery; so this type of therapeutic method may be the cause of her new onset headaches. There are a few reports of headache exacerbation following the gamma knife radiosurgery.^8^

According to the recent studies, post gamma knife headaches are rare, but with an increasing number of gamma knife surgeries, the estimation of the true frequency of gamma knife-induced headaches seems to be important.^8^ Also the severity of the reported headaches makes the detecting and explaining of post-gamma knife headache syndrome very fundamental, and can lead to the best treatment plans by finding the possible etiology of them.

Intractable headache with autonomic features following gamma knife radiosurgery as in our patient, representing a new concern which will be important in case of diagnosis and treatment especially with an increasing number of gamma knife surgeries.

## Notes:

**How to cite this article:** Togha M, Naser Moghadasi A, Haghighi S, Motamedi M. Intractable Headache with Autonomic Features after Gamma Knife Radiosurgery: A Case Report. Iran J Neurol 2019; 18(3): 143-4.
